# Early reduction in tumour [^18^F]fluorothymidine (FLT) uptake in patients with non-small cell lung cancer (NSCLC) treated with radiotherapy alone

**DOI:** 10.1007/s00259-013-2632-3

**Published:** 2014-02-07

**Authors:** Ioannis Trigonis, Pek Keng Koh, Ben Taylor, Mahbubunnabi Tamal, David Ryder, Mark Earl, Jose Anton-Rodriguez, Kate Haslett, Helen Young, Corinne Faivre-Finn, Fiona Blackhall, Alan Jackson, Marie-Claude Asselin

**Affiliations:** 1 Institute of Population Health, Wolfson Molecular Imaging Centre, Manchester Academic Health Sciences Centre, The University of Manchester, Manchester, M20 3LJ UK; 2 Manchester Cancer Research Centre, Manchester Academic Health Sciences Centre, The University of Manchester, Manchester, M20 4BX UK; 3 The Christie NHS Foundation Trust, Wilmslow Road, Manchester, M20 4BX UK; 4 AstraZeneca Pharmaceuticals, Alderley Park, Macclesfield, SK10 4TG UK

**Keywords:** [^18^F]Fluorothymidine PET, Non-small cell lung cancer, Radiotherapy, Early response monitoring

## Abstract

**Purpose:**

Changes in tumour 3′-deoxy-3′-[^18^F]fluorothymidine (FLT) uptake during concurrent chemo-radiotherapy in patients with non-small cell lung cancer (NSCLC) have been reported, at variable time points, in two pilot positron emission tomography (PET) studies. The aim of this study was to assess whether FLT changes occur early in response to radiotherapy (RT) without concurrent chemotherapy and whether such changes exceed test-retest variability.

**Methods:**

Sixteen patients with NSCLC, scheduled to have radical RT, underwent FLT PET once/twice at baseline to assess reproducibility and/or after 5–11 RT fractions to evaluate response. Primary and nodal malignant lesions were manually delineated on CT and volume, mean and maximum standardized uptake values (SUV_mean_ and SUV_max_) estimated. Analysis included descriptive statistics and parameter fitting to a mixed-effects model accounting for patients having different numbers of evaluable lesions.

**Results:**

In all, 35 FLT PET scans from 7 patients with a total of 18 lesions and 12 patients with a total of 30 lesions were evaluated for reproducibility and response, respectively. SUV_mean_ reproducibility in primary tumours (SD 8.9 %) was better than SUV_max_ reproducibility (SD 12.6 %). In nodes, SUV_mean_ and SUV_max_ reproducibilities (SD 18.0 and 17.2 %) were comparable but worse than for primary tumours. After 5–11 RT fractions, primary tumour SUV_mean_ decreased significantly by 25 % (*p* = 0.0001) in the absence of significant volumetric change, whereas metastatic nodes decreased in volume by 31 % (*p* = 0.020) with a larger SUV_mean_ decrease of 40 % (*p* < 0.0001). Similar changes were found for SUV_max_.

**Conclusion:**

Across this group of NSCLC patients, RT induced an early, significant decrease in lesion FLT uptake exceeding test-retest variability. This effect is variable between patients, appears distinct between primary and metastatic nodal lesions, and in primary tumours is lower than previously reported for concurrent chemo-RT at a similar time point. These results confirm the potential for FLT PET to report early on radiation response and to enhance the clinical development of novel drug-radiation combinations by providing an interpretable, early pharmacodynamic end point.

**Electronic supplementary material:**

The online version of this article (doi:10.1007/s00259-013-2632-3) contains supplementary material, which is available to authorized users.

## Introduction

Lung cancer is the most common cause of cancer-related mortality worldwide [1]. Radiotherapy (RT) plays a major therapeutic role in patients with non-small cell lung cancer (NSCLC). RT is given with radical intent in patients with early-stage disease who are medically unfit for resection and in locally advanced inoperable disease [2]. Patients with locally advanced (stage III) NSCLC are generally treated with a combination of chemotherapy and RT delivered sequentially or concurrently [3]. Although concurrent chemo-RT has been shown to be associated with improved outcomes this is at a cost of added toxicity and utilized in empirically selected patients, underlining the need for robust efficacy and toxicity biomarkers in both approaches [4].

Further improvements in outcome will require the use of minimally invasive response biomarkers in two distinct but interdependent settings: Firstly, in the preclinical and early-phase clinical trial setting where the inclusion of surrogate pharmacodynamic end points, measured at the population level, may facilitate the rational selection and optimization of novel RT-based regimens [5, 6] and secondly, in late-phase clinical trials and, potentially, in routine clinical practice where response stratification across individual subjects and/or lesions may enable spatially and/or temporally adaptive radiotherapeutic approaches [7, 8]. As a prerequisite step further technical validation and qualification of candidate biomarkers is required [9].

Intrinsic radiosensitivity and tumour repopulation are (along with cell **r**epair, **r**e-oxygenation and cell cycle **r**edistribution) two of the five “R’s” determining RT efficacy according to classical radiobiology [10]. As both are intimately linked to changes of tumour proliferation, it is reasonable to hypothesize that monitoring changes of tumour proliferation during RT may provide surrogate information on response and patient outcome.

3′-deoxy-3′-[^18^F]Fluorothymidine (FLT) positron emission tomography (PET) allows non-invasive evaluation of tumour proliferation. FLT uptake primarily depends on phosphorylation mediated by thymidine kinase 1 (TK1). TK1 is up-regulated 10- to 20-fold during S phase; therefore, FLT uptake parameters provide a surrogate measurement for S-phase fraction and proliferation rate [11]. Validation studies have demonstrated significant correlations between baseline lung tumour FLT uptake parameters and the proliferation index Ki-67 [12–16].

In xenograft models, irradiation induced early decreases of tumour FLT uptake, generally preceding [^18^F]fluorodeoxyglucose (FDG) changes [17–20]. Two pilot studies have reported changes of tumour FLT uptake at variable time points during the course of concurrent/sequential chemo-RT, each including five patients with NSCLC [21, 22]. In the two patients who were studied after 2 days of treatment, primary tumour maximum standardized uptake value (SUV_max_) exhibited changes of +22 and −27 %. After 8 days larger decreases of 39 and 40 % were measured in two patients [21]. The remaining patients were studied in the fifth week of treatment and large variable decreases were reported in primary tumour SUV_max_, ranging from 25 to 71 %, after 44–48 Gy [22]. Although the first study reported “a disparity in the anatomical and metabolic response of primary tumour and lymph nodes” [21], no statistically significant differences were found in FLT responses between the two lesion types in the second [22]. As most patients studied received concurrent chemo-RT, the relative contribution of the two treatment components to the observed changes is unclear. To date there are no data available relating to FLT uptake changes in response to RT alone.

We conducted a prospective study aiming to investigate the magnitude, significance and variability of tumour FLT uptake changes after 1–2 weeks of radical RT without concurrent chemotherapy. This work was performed as a sub-study of RADAR (Radiation Damage And Resistance in lung cancer), a Manchester Lung Cancer Group multimodality study aiming to discover, evaluate and validate novel blood and imaging biomarkers of RT response and toxicity in lung cancer.

## Materials and methods

### Patients

We recruited patients with a histological or cytological diagnosis of NSCLC scheduled to receive radical RT as per standard of care. Patients who had received induction chemotherapy prior to RT were eligible; however, those planned to have concurrent chemo-RT were excluded. Other eligibility criteria included Eastern Cooperative Oncology Group (ECOG) performance status 0 or 1, presence of a primary tumour exceeding 2 cm in maximum diameter and written, informed consent. In women of childbearing potential, pregnancy was excluded by measurement of urine beta human chorionic gonadotropin (β-HCG). The study protocol was approved by the local Research Ethics Committee (REC, 09/H1011/55) and Administration of Radioactive Substances Advisory Committees (ARSAC, 595-3742/25138) and has therefore been performed in accordance with the ethical standards laid down in the 1964 Declaration of Helsinki and its later amendments.

Patients were treated on a linear accelerator using 6-MV photons with a total dose of 50–55 Gy in 20 once-daily fractions or 60–66 Gy in 30–33 fractions. Consenting patients underwent one or two (optional) baseline FLT PET/CT scans (Scan1/Scan2) followed by a further scan during the second week of treatment (Scan3). Therefore, patients were evaluable for either baseline reproducibility of FLT PET parameters, RT response or both.

The individual time intervals between scans and treatments are tabulated in Table 1. Patients who had received induction chemotherapy underwent the first baseline scan 11–49 days (median 26 days) after the last cycle. In reproducibility-evaluable patients, baseline scans were performed within 2–6 days (median 4.0 days) of each other. In response-evaluable patients, RT was initiated 1–13 days after the latest/single baseline scan (median 5.0 days). Response assessment scans were performed after 6–15 calendar days on treatment (median 8.5 days), corresponding to 5–11 RT fractions (median 7.5 fractions).

**Table 1:**
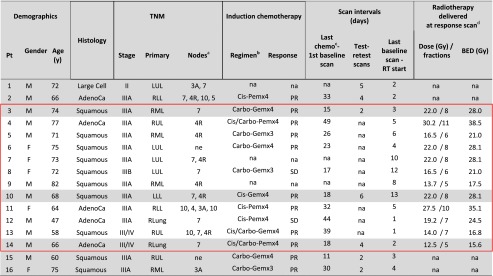
Patient (Pt) characteristics. *TNM* tumour, node, metastasis staging, *AdenoCa* adenocarcinoma, *LUL* left upper lobe, *RLL* right lower lobe, *RML* right middle lobe, *LLL* left lower lobe, *RUL* right upper lobe, *PR* partial response, *SD* stable disease, *BED* biologically effective dose, *na*, non-applicable, *Cis* cisplatin, *Pem* pemetrexed, *Carbo* carboplatin, *Gem* gemcitabine. Reproducibility-evaluable patients are highlighted in *grey* and response-evaluable patients are encompassed within *red* *border*

^a^ International Association for the Study of Lung Cancer lymph node map (2009). ^b^ / indicates drug switch, - denotes drug combination and x precedes number of chemotherapy cycles. ^c^ Date of last chemotherapy administration. ^d^ All patients in the response cohort were treated with a total dose of 50-55 Gy in 20 once-daily fractions except one patient (13) who was given 66 Gy in 33 fractions.

Clinical follow-up included medical history and physical examination 1, 3 and 6 months following completion of RT and subsequently as per local practice. Imaging follow-up included contrast-enhanced CT of thorax and abdomen at 4 weeks, 6 months and 12 months following completion of RT and subsequently as required at the discretion of the treating physician. Overall survival (OS) was defined as time from RT initiation to death, irrespective of cause. Locoregional control (LRC) was defined as time from RT initiation to local disease progression according to RECIST 1.1 criteria [23]. For patients that are still alive and/or local progression had not been detected at the time of death, LRC and OS were calculated relative to the date of last follow-up.

### PET data acquisition

FLT was either synthesized in-house as described in [24] or externally supplied (PETNET Solutions Inc., Nottingham, UK or Wolfson Brain Imaging Centre, University of Cambridge, Cambridge, UK).

All PET scans were performed at the Wolfson Molecular Imaging Centre (WMIC, Manchester, UK) using the Biograph 6 Truepoint TrueV scanner (Siemens Molecular Imaging Inc., Knoxville, TN, USA). This is a four-ring lutetium oxyorthosilicate (LSO)-based 3D PET scanner with an axial field of view (FOV) of 21.6 cm coupled with a six-slice helical CT scanner. FLT was injected as a 30-s bolus of 254–361 MBq (mean 311 MBq), and each emission scan was performed at a single bed position without respiratory gating. The PET scan was followed by a non-breath-hold, non-contrast-enhanced CT scan according to manufacturer recommendations (peak energy 130 keV, 70 mAs). CT data were reconstructed at high and low spatial resolutions for volumes of interest (VOI) delineation and for attenuation and scatter correction purposes, respectively. PET data acquired 45–60 min post-injection were reconstructed as a single frame using 3D ordered subset expectation maximization (OSEM) with 4 iterations and 21 subsets to a 256 × 256 × 109 matrix, corresponding to voxel size of 2.67 × 2.67 × 2.00 mm^3^. PET images were smoothed using a 3D 4-mm Gaussian filter post-reconstruction.

### Image analysis

PET images were decay-corrected and normalized for injected dose and patient body weight using Eq. 1 to generate parametric SUV maps:

1 $$ SUV\left(\frac{g}{ ml}\right)= Tissue\; radioactivity\left(\frac{ kBq}{ ml}\right)\times \frac{ Weight(kg)}{ Injected\; dose(MBq)} $$

**Fig. 1:**
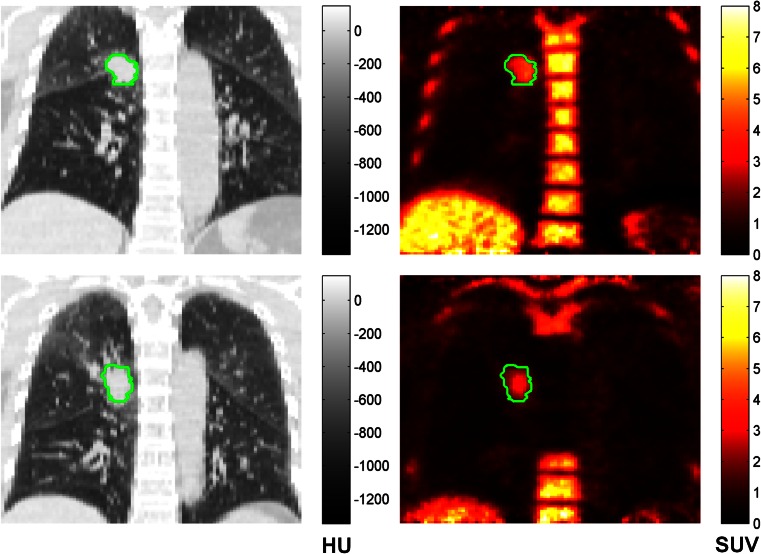
Representative CT (*left*) and PET (*right*, SUV 45–60 min) images of patient 11 acquired at baseline (*top*) and after delivery of 27.5 Gy (*bottom*). Primary tumour is manually delineated within *green outline*. Note sharply demarcated dramatic reduction in bone marrow uptake and modest reduction of tumour uptake after RT, as previously reported [21]

Primary tumours and metastatic lymph nodes were manually delineated slice by slice transaxially by an experienced oncological radiologist (B.T.) to generate 3D VOIs on the baseline and response scans using Analyze image analysis software (Biomedical Imaging Resource, Mayo Foundation, Rochester, MN, USA). Lesion delineation aimed to include entire malignant lesions as whole, potentially heterogeneous anatomical entities and was not limited to FLT-avid areas. Hence VOI delineation was primarily CT-based and co-registered PET images were only considered as required, for example to aid in the delineation of tumour from consolidated lung or to more accurately determine the margins of masses which abutted the mediastinum. Standardized CT window settings were used to delineate tumour from adjacent lung (lung windows; level −500 HU, width 1,500 HU) or from adjacent soft tissues (soft tissue windows; level 0 HU, width 400 HU). A fixed visualization window between 0 and 4 was used for the smoothed SUV maps. Enlarged mediastinal nodes or nodes of normal size showing increased FLT uptake were included in the analysis and delineated on CT with soft tissue windows. Representative CT and PET SUV images of patient 11 are shown in Fig. 1 at baseline and after 10 RT fractions. Contamination of tumour VOIs by normal proliferating bone marrow signal may cause substantial errors such that in tumours adjacent to the skeleton particular care was taken to ensure a margin between the delineated VOI and bone. The quality of PET/CT co-registration, which can be compromised by patient movement, was visually assessed and VOIs were manually adjusted as required.

### Statistical analysis

For each evaluable VOI (primary tumour or metastatic lymph node), three parameters were extracted: volume in cm^3^, SUV_mean_ and SUV_max_. Individual test-retest difference and RT-induced response were calculated using Eqs. 2 and 3, respectively:

2 $$ Test- retest\  difference\ \left(\%\right)=\frac{ Scan2- Scan1\ }{ Scan1+ Scan2}\times 200 $$

3 $$ Response\ \left(\%\right)=\frac{ Scan3- Baseline\ }{ Baseline}\times 100 $$

where *Baseline* is the latest/single baseline parameter value for *Scan1* or *Scan2*. Reproducibility was estimated as the standard deviation (SD) of the mean test-retest differences across patients and as the average of the absolute test-retest differences across patients (absolute reproducibility).

To allow comparison of the effects of different fractionation regimens, we calculated the biologically effective dose (BED) at the time of the response scan (Scan3) using Eq. 4:

4 $$ BED=n\times D\times \left(1+D/\left(\frac{a}{\beta}\right)\right) $$

where *n* is the number of RT fractions, *D* (in Gy) is the administered dose per fraction and *α/β* was set to 10 for both primary and nodal lesions [25].

#### Descriptive statistics

Descriptive summary statistics of the difference between Scan1 and Scan2 for each parameter including mean and SD were calculated. The repeatability coefficient (RC = 1.96 × SD) was also calculated to support comparison with previously published reproducibility values for FLT [26, 27]. Differences between lesion volume and SUV before and during RT were tested using the Wilcoxon rank sum test. Potential correlations amongst baseline parameters and between baseline parameter values and reproducibility values were assessed using Spearman’s rho coefficient test (two-sided). No correction for multiple testing was used in this exploratory analysis.

#### Mixed-effects models

As most patients had more than one evaluable lesion, the SUV_mean_, SUV_max_ and volume values, transformed to a common (base 10) logarithmic scale for the purpose of this analysis, were each fitted separately to two linear mixed-effects models using analysis of covariance (ANCOVA): The first model (A) pertains to the reproducibility-evaluable patients and was implemented on the duplicate baseline values with the Scan2 value representing the dependent variable. Covariates included the Scan1 value and type of lesion (primary vs node) as fixed-effect terms. The second model pertains to the response-evaluable patients and was implemented with the Scan3 value representing the dependent variable. Covariates included the latter/single baseline value (Scan1/2) and type of lesion (primary vs node) as fixed-effect terms. BED was also assessed as an additional covariate. To account for patients having more than one evaluable lesion, a random effect term was included in both models to account for potential nested effects within patients, i.e. heterogeneity across patients. The models are described in more details with the results presented in Table 3.

#### Analysis of outcome data

Median times for LRC and OS were calculated using the Kaplan-Meier method. Potential associations of both outcome end points with primary tumour continuous PET parameters (baseline, on-RT and response values for SUV_mean_ and SUV_max_) were investigated using univariate Cox regression without correction for multiple testing.

Statistical tests were performed using SPSS version 19 (SPSS Inc., Chicago, IL, USA) or the statistical software R. A *p* value below 5 % was considered to be statistically significant.

## Results

Between June 2010 and August 2012, 19 patients were recruited into the study. Three patients withdrew after the first baseline scan and were therefore not evaluable. Of the remaining 16 patients, 3 underwent 2 baseline and 1 on-treatment scans and were evaluable for both reproducibility and response. Four patients underwent two baseline scans and were evaluable for reproducibility only. Nine patients underwent a single baseline and an on-treatment scan and were evaluable for response only. Overall, 7 patients with a total of 7 primary and 11 lymph node lesions were evaluable for reproducibility and 12 patients with a total of 12 primary and 18 lymph node lesions were evaluable for response.

**Fig. 2:**
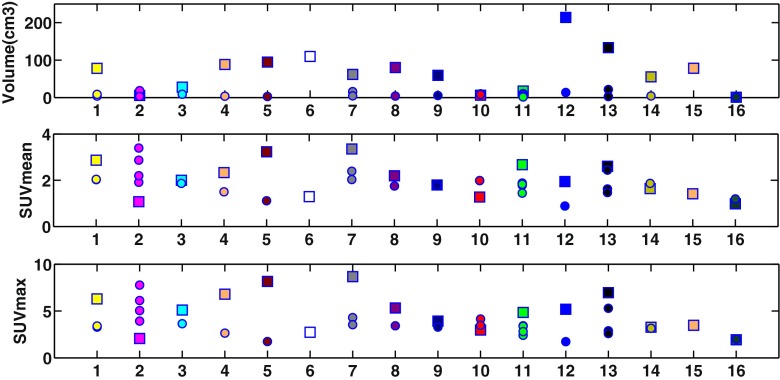
Baseline (single/average) volume (top), SUV_mean_ (middle) and SUV_max_ (bottom) of primary tumours (*squares*) and metastatic nodal lesions (*circles*) of 16 patients

Clinical characteristics of evaluable patients and information on the timing of FLT PET scans are summarized in Table 1. Most subjects had stage III disease (13 of 16) and most (13 of 16) received induction chemotherapy; of the latter, the majority (11 of 13) demonstrated partial response to treatment. Baseline volume, SUV_mean_ and SUV_max_ of all evaluable lesions are shown in Fig. 2. Respective summary statistics of baseline, reproducibility and response values for primary tumours and lymph nodes from the PET/CT images are given in Table 2. The primary tumour volumes were significantly larger (5.0–210.7 cm^3^) than nodal volumes (1.3–21.4 cm^3^) at baseline (*p* < 0.001). Review of lesion SUVs reveals substantial variability of baseline uptake between patients, e.g. primary tumour SUV_mean_ ranged from 0.9 to 3.4 with similar variability observed in nodes (0.9–3.1). In several cases, substantial uptake differences were observed within subject: In 13/16, subjects’ primary tumour uptake was higher than or similar to that of metastatic nodes, whereas in 3 subjects (patients 2, 10 and 16), nodal uptake was greater than the primary tumour.

**Table 2:** Summary statistics of lesion imaging parameters (SUV_mean_, SUV_max_ and volume) with reproducibility and response 1–2 weeks after RT initiation. Values are presented as mean ± SD. For the reproducibility cohort, 7 primaries and 11 nodes are averaged. For the response cohort, 12 primaries and 18 nodes are averaged

Lesion	Reproducibility cohort		Response cohort		Test-retest differences (%)^b^	Responses (%)
	1st baseline values	2nd baseline values	Baseline values^a^	Response values		
Volume (cm^3^)						
Primaries	38 ± 31	40 ± 31	78 ± 56	85 ± 63	9.5 ± **12.8** (**9.8**)	**6.0** ± 19.1
Nodes	7.5 ± 4.0	8.6 ± 6.3	7.4 ± 5.3	5.9 ± 5.5	8.4 ± **22.5** (**19.3**)	**−16.9** ± 57.0
All	19 ± 24	21 ± 25	36 ± 49	38 ± 56	8.8 ± **18.9** (**15.6**)	**−7.7** ± 46.7
SUV_mean_						
Primaries	1.6 ± 0.7	1.6 ± 0.6	2.2 ± 0.7	1.6 ± 0.4	−2.9 ± **8.9** (**6.8**)	**−24.3** ± 13.9
Nodes	2.1 ± 0.5	2.2 ± 0.7	1.7 ± 0.4	1.1 ± 0.4	0.6 ± **18.0** (**11.9**)	**−39.7** ± 14.6
All	1.9 ± 0.6	1.9 ± 0.7	1.9 ± 0.6	1.3 ± 0.5	−0.8 ± **14.9** (**9.9**)	**−33.5** ± 16.0
SUV_max_						
Primaries	3.5 ± 1.7	3.6 ± 1.5	5.3 ± 2.0	4.1 ± 1.4	5.7 ± **12.6** (**9.5**)	**−22.9** ± 13.6
Nodes	4.0 ± 1.3	4.3 ± 1.9	3.2 ± 0.9	1.8 ± 0.7	3.1 ± **17.2** (**14.0**)	**−44.4** ± 15.9
All	3.8 ± 1.5	4.1 ± 1.8	4.1 ± 1.7	2.7 ± 1.5	4.1 ± **15.3** (**12.3**)	**−35.8** ± 18.2

^a^The second baseline scan was used for patients with two baseline scans

^b^Reproducibility given by the SD of the mean test-retest differences. Average of the individual absolute test-retest differences added in parentheses. Both estimates and mean responses highlighted in bold

Across all lesions, there was a strong significant correlation between baseline SUV_max_ and SUV_mean_ (Spearman’s rho 0.91, *p* < 0.001) and a modest but also significant correlation between SUV_max_ and volume (rho 0.58, *p* < 0.001), whereas a weak significant correlation was found between SUV_mean_ and volume (rho 0.36, *p* = 0.020). When primary tumours were examined separately from nodes, SUV_max_ correlated significantly with SUV_mean_ for both lesion types (primary tumours rho 0.93, *p* < 0.001; nodes rho 0.92, *p* < 0.001). Likewise, SUV_max_ correlated significantly with volume (primary tumours rho 0.59, *p* = 0.016; nodes rho 0.53, *p* = 0.006), but SUV_mean_ correlated significantly with volume only for the nodes (primary tumours rho 0.35, *p* = 0.19; nodes rho 0.46, *p* = 0.020).

**Fig. 3:**
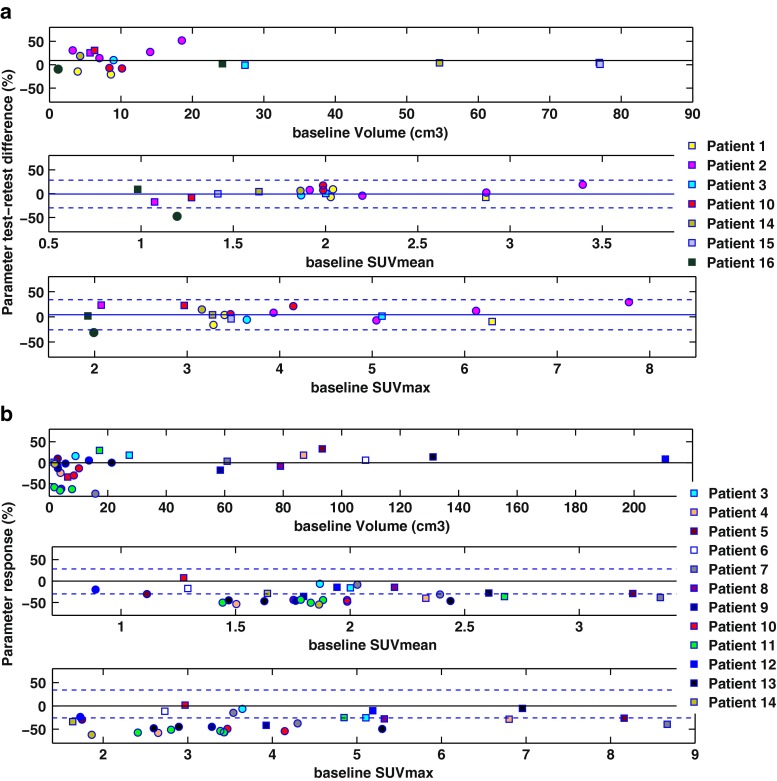
**a** Lesion reproducibilities (%) plotted against mean baseline lesion values for volumes (*top*), SUV_mean_ (*middle*) and SUV_max_ (*bottom*). **b** RT-induced responses (%) plotted against baseline parameter values (second baseline scan if two baseline scans acquired). *Dashed lines* in SUV graphs set at mean test-retest difference ± RC = 1.96 × SD, where SD is standard deviation of respective parameter reproducibility. In volume graphs *dashed lines* not displayed as absolute volume reproducibility correlated with baseline average volume. Primary tumours represented by *squares* and metastatic nodes by *circles*. The second evaluable lesion of patient 7 (4R) was measured as 170 % larger post-RT. This outlier is not shown in **b**, to enhance visibility, nevertheless was included in the statistical analyses

Reproducibility of individual patients is plotted in relation to baseline values in Fig. 3a. Across all lesions, the absolute SUV test-retest differences did not correlate with average baseline lesion SUV_max_ (rho −0.10, *p* = 0.70) or SUV_mean_ (rho −0.10, *p* = 0.69). In contrast, we found a significant negative correlation between absolute volume test-retest differences and average lesion volume (rho −0.57, *p* = 0.015). Accordingly, primary tumour volume reproducibility (SD 12.8) was substantially better compared to nodal lesions (SD 22.5). Consequently, volume RCs were estimated separately for these lesion types, whilst the SUV RC was estimated jointly for primary tumour and nodal lesions and was similar for SUV_mean_ (SD 14.9 %) and SUV_max_ (SD 15.3 %).

RT response of individual patients is similarly plotted against baseline values in Fig. 3b. RT-related primary tumour volume changes ranged from −33.9 to + 33.1 % (mean change +6 %, *p* = 0.84). In most (9/12) cases, these changes did not exceed the RC (25 %). On the other hand, metastatic node volume changes ranged from −90.0 % to an outlier increase of +170.4 %, demonstrating a decrease exceeding the RC (44 %) in 5/18 lesions from 3/12 patients. RT-induced changes of lesion SUVs ranged from −54.9 to +7.6 % for SUV_mean_ and from −62.0 to +1.6 % for SUV_max_. Examined separately, nodes demonstrated a 40 ± 15 % decrease in SUV_mean_ that on average was 1.6 times higher than the 24 ± 14 % decrease of primary tumours (1.9-fold for SUV_max_). SUV_mean_ response correlated strongly with SUV_max_ response (rho 0.89, *p* < 0.0001).

**Fig. 4:**
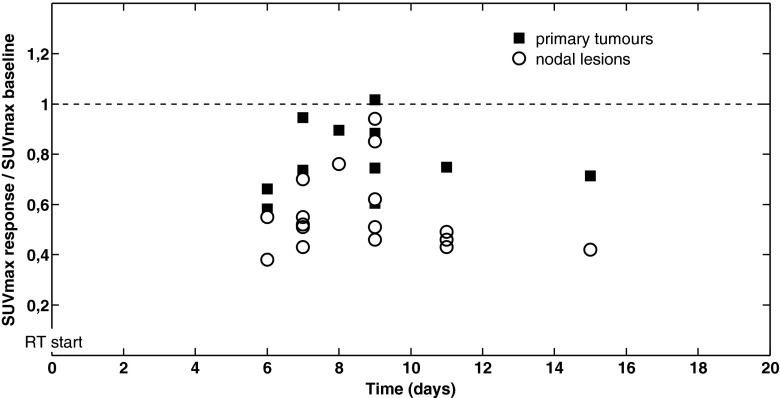
Lack of dose-response relationship between SUV_max_ response (expressed as ratio of SUV_max_ during RT to single/latest baseline value) and calendar days on RT. Primary tumours represented by *squares* and metastatic nodes by *circles*

Malignant lesion SUV_max_ during RT (normalized to single/latest baseline value) is plotted against calendar days on RT in Fig. 4, as previously presented by Everitt et al. [21]. During the course of our response assessment window, no significant dose-response relationship was found between SUV_max_ response in primary tumours and days since start of RT (rho = 0.27, *p* = 0.39), nor with RT dose delivered or BED.

The results of the ANCOVA are outlined in Table 3. The mixed-effects model implemented on the reproducibility data set (model A) did not provide any evidence of a systematic effect (progression/decline) of the retest values in relation to the test values for any of the parameters SUV_max_, SUV_mean_ or volume for either primaries or nodes. The fit of the response data to model B did not improve by adding BED as a covariate for any of the parameters SUV_max_, SUV_mean_ or volume. Therefore, BED was not included in the final implementation of model B. On average, the model estimated a significant decrease in SUV_mean_ of 25 % [confidence interval (CI) −15 to −34 %] in the primary lesions post-RT. Furthermore, the treatment effect for nodes was significantly different to primaries (*p* = 0.004), with a larger 40 % (CI −33 to −46 %) decrease. Similarly, the model estimated a significant RT-related decrease of primary tumour SUV_max_ by an average of 24 % (CI −13 to −34 %), which was significantly lower (*p* = 0.0006) compared to the average 46 % (CI −39 to −52 %) decrease in SUV_max_ for nodes.

**Table 3:** Tabulated results of ANCOVA performed separately for lesion SUV_mean_, SUV_max_ and volume at duplicate baseline scans (model A) and in response to RT (model B). The linear mixed-effects model was generally formulated as *log*10(*y*_1*ij*_) = *log*10(*y*_0*ij*_) + (*β*_0_ + *b*_*i*_) + *β*_1_ *x*_1*ij*_ + *ε*_*ij*_. In model A, *y*_0*ij*_ is the initial baseline and *y*_1*ij*_ is the second baseline value for patient *i* and lesion *j*, whereas in model B, *y*_0*ij*_ is the pre-RT value and *y*_1*ij*_ is the post-RT value for patient *i* and lesion *j*. In both models, *x*_1_ indicates the type of lesion (set to 0 if primary or 1 if node) such that *β*_0_ is the ‘intercept’ for primaries and *β*_0_ + *β*_1_ is the intercept for nodes. In both models, *b*_*i*_ ∼ *N*(0,  *σ*_*b*_^2^) represents the cross-patient heterogeneity random term (σ_b_^2^ is the variance across patients) and *ε*_*ij*_ ∼ *N*(0,  *σ* ^2^) represents the model error that is due to variability across lesions and ‘pure’ model error (σ^2^ is the residual error variance). Note that parameter estimates are based on log-transformed data and hence need to be back-transformed to allow estimation of effect size. For example, the model-estimated mean RT-induced SUV_mean_ effect size ratio can be calculated from *β*_0_, *β*_1_ values from model ${y}_1/{y}_0-1={10}^{\left({\beta}_0+{\beta}_1\right)}-1$ for nodes. The proportion of the variation in the intercept that is accounted for by patient heterogeneity can be calculated as *σ*_*b*_^2^/(*σ* ^2^ + *σ*_*b*_^2^). The Akaike Information Criterion (AIC) provides a measure of the goodness of the fit to the four-parameter ($\left({\beta}_0,{\beta}_1{,}_{{\sigma_{\mathrm{b}}}^2,{\sigma}^2}\right)$ models

Model A (reproducibility) parameters		SUV_mean_	SUV_max_	Volume
Fixed effects^a^	β_0_^b^	−0.014 ± 0.025 (0.60)	0.024 ± 0.026 (0.37)	0.041 ± 0.031 (0.11)
	β_1_^c^	0.015 ± 0.032 (0.65)	−0.025 ± 0.027 (0.38)	−0.025 ± 0.031 (0.43)
Random effects	σ_b_^2^	0.00000	0.00188	0.00300
	σ^2^	0.00448	0.00291	0.00374
AIC		−28.78	−30.20	−25.42
Model B (response) parameters		SUV_mean_	SUV_max_	Volume
Fixed effects^a^	β_0_^b^	−0.127 ± 0.025 (0.0001)	−0.119 ± 0.029 (0.0007)	0.018 ± 0.073 (0.81)
	β_1_^c^	−0.095 ± 0.029 (0.004)	−0.147 ± 0.035 (0.0006)	−0.177 ± 0.089 (0.06)
Random effects	σ_b_^2^	0.00224	0.00154	0.00858
	σ^2^	0.00556	0.00839	0.05579
AIC		−45.24	−37.02	15.46

^a^Model parameters for fixed effects presented as estimate ±standard error (*p* value)

^b^ *p* values from statistical comparison against 0

^c^ *p* values from statistical comparison against primary tumours

With regard to lesion volume, primary tumours demonstrated no significant change (4 %, CI −27 to +48 %, *p* = 0.81) in response to treatment. In contrast, the model estimated an average decrease of 31 % (CI −6 to −49 %) in node volume that was significant (*p* = 0.020) and significantly different to primary tumours (*p* = 0.060).

For all three parameters, the random variation across patients estimated by model B was small compared to the variation across lesions. For SUV_mean_, the cross-patient (nested) effect accounted for about 28.7 % of the total variance. The contribution of cross-patient variance to the total variance was even smaller for SUV_max_ and volume at 15.5 and 13.3 %, respectively. One year after recruiting the last patient (September 2013), the median follow-up time was 15.4 months after RT initiation (range 3–33 months). At that time point, 9 patients had documented local primary tumour progression, 5 had developed metastatic disease and 11 had died of which 1 (patient 2) before treatment completion. Median LRC and OS times were 12.2 (CI 6–18) and 19.5 (CI 9–30) months, respectively. Cox regression analysis identified no significant association between OS and baseline, on-RT or response values of primary tumour SUV_mean_ and SUV_max_ (*p* > 0.08). However, LRC was found to be associated with primary tumour SUV_max_ on-RT (hazard ratio 2.31, CI 1.06–5.00, *p* = 0.034) but not with SUV_max_ at baseline (hazard ratio 1.46, CI 0.97–2.18, *p* = 0.068) or SUV_mean_ on RT (hazard ratio 5.80, CI 0.66–50.6, *p* = 0.112). This exploratory analysis is graphically presented for LRC in Supplementary Fig. 1.

## Discussion

We have confirmed previous studies on FLT parameter reproducibility and demonstrated that, in patients with NSCLC, RT induces an early, significant decrease in tumour FLT uptake which is lower than previously reported for concurrent chemo-RT at a similar time point [21]. After 1–2 weeks of RT, the magnitude of this response was found not to be driven by variations in the RT dose delivered but to vary between primary tumours and metastatic nodal lesions, possibly indicating distinct intrinsic radiosensitivities. This contrasts with the previously reported findings that FLT response did not to differ between lesion types after 5–6 weeks of chemo-RT and depended on the RT dose received [22].

Establishing the effect of treatment on a potential imaging biomarker is best performed in the context of validating its precision. Therefore, we performed a combined assessment of tumour FLT PET parameter reproducibility and response. To date, three groups have reported on the reproducibility of tumour FLT SUVs in patients with lung and other cancers [28–30], while two further publications addressed the issue of metabolic volume reproducibility [26, 27]. In all cases, tumour VOIs were delineated on PET images, either manually or using semi-automatic segmentation methods. In contrast, in our study, tumour VOIs were delineated primarily on anatomical CT images, aiming to capture the mean uptake across both avid and less avid tumour regions. Furthermore, previous studies largely included treatment-naive subjects, whereas most patients enrolled in our study had received induction chemotherapy before acquisition of the baseline scans. Consequently, average lesion SUV_mean_ in our subjects was substantially lower than in previous studies (SUV_mean_ ∼ 2 compared to more than 3). Despite such differences, SUV_mean_ reproducibility results across studies were comparable: Reproducibility in our study was 14.9 % (primaries 8.9 %, nodes 18.0 %) compared to 10.5 % [28], 7.0 % [29] and 4.5 % [30] in previous reports exclusively or predominantly including primary lesions. Despite the use of image smoothing and absence of variability associated with VOI delineation, primary tumour SUV_max_ was less reproducible (SD 12.6 %) than SUV_mean_. These results are in agreement with the main conclusion of a recent meta-analysis of FDG studies demonstrating SUV_mean_ as the most reproducible SUV parameter [31]. This superiority was lost in nodal lesions for which SUV_max_ was marginally more reproducible than SUV_mean_.

In terms of volume reproducibility, we found that manual delineation of malignant NSCLC lesions by a single imaging expert yielded VOIs with volumetric precision at least equivalent to semi-automatic segmentation methods including fixed threshold, adaptive threshold or cluster-based methods implemented in two previous publications in patients with lung and breast cancer [26, 27]. However, it is expected that manual tumour delineation, unlike semi-automatic methods, will suffer from additional inter-observer variability [32]. In accordance with previous reports, we found that the volume reproducibility of manually delineated lesions correlated negatively with their size [26, 27]. Therefore, using the same confidence intervals across a wide spectrum of volumes is inappropriate. Optimal volumetric response assessment would require one or more cut-off values defined through analysis of a large reproducibility data set and/or simulation studies.

Taken together, this and other recent reports indicate that, in lung cancer, FLT SUV parameters are similarly robust to respective FDG parameters, with only a modest impact from the segmentation approach used. Changes of primary tumour SUV_mean_ exceeding 17 % and SUV_max_ exceeding 25 % are likely to be significant. Lesion volume, delineated manually on PET/CT images or with semi-automatic PET-based segmentation methods is generally a less precise parameter, especially for small lesions.

When we designed this study, we considered the choice of potential time points to assess response. Based on preclinical reports [17–20] and the first pilot clinical study [21], we elected an early assessment point aiming to obtain an early read-out of intrinsic radiosensitivity and minimize the confounding effect of volumetric changes and potential RT-related inflammatory changes on tumour uptake. Furthermore, to limit the variability of RT dose delivered at the time of on-treatment scans, while maintaining study feasibility, we restricted the response assessment window to 1 week.

We found that in the absence of significant changes in average primary tumour size, RT induced a significant decrease of about 25 % in FLT uptake after a week of treatment, as measured by both SUV_mean_ and SUV_max_. In contrast, metastatic nodes sustained a significant decrease in mean volume associated with a decrease in FLT uptake significantly exceeding that observed in primary tumours. Considering that primary tumours routinely receive RT doses at least equivalent to metastatic nodes, the observed difference in uptake response may be attributed to genuine differences in biological behaviour between primary and metastatic nodal lesions in terms of perfusion, FLT-specific transport and/or phosphorylation in response to treatment. These processes cannot be separated by simple SUV measurements. Alternatively, the RT dose delivered to the metastatic nodes may differ from that to the primary tumour. Finally, considering the potential interaction of size and uptake, which may be enhanced in small/shrinking lesions, it is possible that the observed decreases of the size of some nodal lesions may have contributed to an artefactual decrease of measured uptake. On a different but relevant note, response results will differ on whether absolute or percentage differences are calculated. Considering that in most response-evaluable patients baseline primary tumour uptake exceeded that of metastatic nodes, an absolute difference response assessment would attenuate differences in response between primaries and nodes.

In addition to differences between primaries and nodes, we observed variable changes in FLT uptake across all lesions and across primaries and nodes separately. This is complicated further by the fact that different patients had variable numbers of evaluable lesions. To disentangle these interactions, we used a mixed-effects linear model. The statistical outcome suggests that the response variability across patients (i.e. a nested effect) had a limited contribution to the overall variability, which was mainly driven by lesion-to-lesion differences. In other words, response heterogeneity was most prominent across lesions rather than across patients. Variations in the RT dose delivered over the course of a week did not explain the variability in FLT responses in the early phase of treatment. Considering the spectrum of baseline variability and treatment-related changes illustrated in Fig. 3, this point in the course of RT SUV analysis may support early response stratification of lesions into responding and not responding.

Two previous studies have reported treatment-related FLT SUV_max_ changes, each in five patients each receiving concurrent chemo-RT. Everitt et al. were first to assess patients with NSCLC at variable time points ranging from 2 to 29 days of treatment [21]. After 10 Gy, primary tumour SUV_max_ reduced by 40 % in two cases, while in two subjects 20 Gy induced decreases exceeding 60 %, as compared to an average decrease of 24 % after 12–30 Gy in our study. These differences may to some extent reflect the added effect of concurrent chemotherapy. Vera et al. studied NSCLC patients using FLT, FDG and [^18^F]fluoromisonidazole (FMISO for hypoxia) PET at baseline and after 4–5 weeks of radical concurrent/sequential chemo-RT [22]. The decrease in FLT SUV_max_ at this later time point was 46 ± 21 % in primary tumours, comparable to that previously reported by Everitt et al. [21] and appeared lower and more variable (29 ± 60 %) in mediastinal lymph nodes; nevertheless, no significant differences between primaries and nodes were found. In contrast to our results, and despite a narrower response assessment window (44–58 vs 12–30 Gy), they interestingly found that one third of the variations in FLT responses could be accounted by the dose-response relationship when considering tumours and nodes together and adjusting for inter-patient heterogeneity, possibly indicating that during the latter phase of treatment tumour uptake may be undergoing more dynamic changes. Neither lesion volumes nor changes in volume which could have confounded the changes in FLT uptake, especially in the later phase of treatment, were reported in this study.

Similarly to previous investigators [21], we observed a dramatic reduction in FLT uptake of normal bone marrow within the RT field. In this study, at least half of evaluable primary tumours and/or metastatic nodal lesions were adjacent or proximal to proliferating bone. It is worth noting that in such cases there is a risk for incorrect response assessment, which is exacerbated for RT compared to chemotherapy. For example, erroneous inclusion of a segment of proliferating bone marrow within the tumour VOI at baseline may lead to response overestimation. Such a risk may be higher in the absence of co-registered CT, or in cases of PET/CT mis-registration, and can have major effect on study results, in particular when SUV_max_ is used to assess response. We tried to minimize this risk by comprehensive co-registration assessment and exclusion of any overlap of the skeleton with tumour VOIs.

Compared to the concurrent approach, sequential chemo-RT lends itself to the separate study of systemic and local treatment on tumour response. Nevertheless, recent chemotherapy could still have influenced the changes observed on-RT by either affecting the baseline tumour proliferative status or through a persistent pharmacodynamic effect especially in the early course of RT. Although the interval between chemotherapy completion and baseline imaging in response-evaluable subjects was at least 2 weeks and duplicate baseline scans up to a week apart did not detect significant changes in tumour size or proliferative activity, induction chemotherapy could have confounded changes in tumour FLT uptake attributed to RT to a degree not quantifiable in this data set.

Although survival analysis was not a primary objective of this study, we explored potential relationships of primary tumour SUV parameters, at baseline, during treatment and their relative change, with tumour control and patient outcome. Instead of defining arbitrary cut-off point(s) that would split the small sample size into smaller subgroups sensitive to the cut-off point(s) selected, we followed a univariate regression approach. Our results indicate a possible association of primary tumour SUV_max_, but not SUV_mean_, after 1–2 weeks of RT and eventual LRC. On the other hand, OS, potentially influenced by multiple variables in addition to local control, was not significantly associated with any local tumour imaging parameter. This interesting finding should be interpreted with caution because of the lack of detection power and the risk of type I error associated with multiple testing on a small sample size, which was further reduced by censoring. However, it could serve as an a priori hypothesis to be formally tested by a larger subsequent study, ideally with pre-planned multivariate analysis to account for additional clinical and imaging parameters.

Despite a larger number of patients compared to earlier work, our study was still limited in size. In our experience, patient recruitment in this setting can be challenging. Lung cancer patients are often symptomatic, frail or elderly and participation in a molecular imaging study is associated with the burden of additional scans without a potential therapeutic benefit. Comprehensive evaluation of FLT PET in this setting would require recruitment of large numbers of patients to be evaluated at various time points, in order to elucidate both the time course and heterogeneity of responses and repopulation. The logistic limitations related to accomplishing such a project in a single centre raise the importance of standardizing methodology and multi-centre collaborations, more easily achieved using SUV than kinetic analysis. A further limitation of our work arises from the lack of some form of respiratory motion correction. Respiratory motion and to a lesser extent partial volume effects are expected to induce an interaction between size and SUV, resulting in bias especially in small peripheral lesions [33, 34].

In summary, this is the first study demonstrating that RT, as single treatment modality, induces in a subgroup of patients an early, significant decrease of primary lung tumour FLT uptake in the absence of significant mean volumetric changes. Metastatic nodes follow a discrete pattern of change with some early volumetric regression and a larger decrease in FLT uptake. These results confirm the potential for FLT PET to report early on radiation response and to enhance the clinical development of novel drug-radiation combinations by providing an interpretable, early pharmacodynamic end point. The variability of responses observed indicates some potential for patient stratification in future clinical trials evaluating RT.

## Electronic supplementary material

Below is the link to the electronic supplementary material.

Supplementary Fig. 1 Primary tumour locoregional control (LRC) plotted against primary tumour baseline SUV_max_ (*left top*), or SUV_mean_ (*right top*), on-RT SUV_max_ (*left middle*), or SUV_mean_ (*right middle*) and relative SUV_max_ (*left bottom*), or SUV_mean_ response (*right bottom*). Univariate Cox regression model *p* values are attached to each subfigure. *Empty* and *marked circles* represent tumours which progressed and censored cases, respectively. (JPEG 699 kb)

High resolution image (EPS 27.9 kb)
