# Supplementary figures and images for: Early reduction in tumour [18F]fluorothymidine (FLT) uptake in patients with non-small cell lung cancer (NSCLC) treated with radiotherapy alone
Source: Eur J Nucl Med Mol Imaging. 2014 Feb 7;41(4):682–93. doi: 10.1007/s00259-013-2632-3 (PMC3955141; doi:10.1007/s00259-013-2632-3)

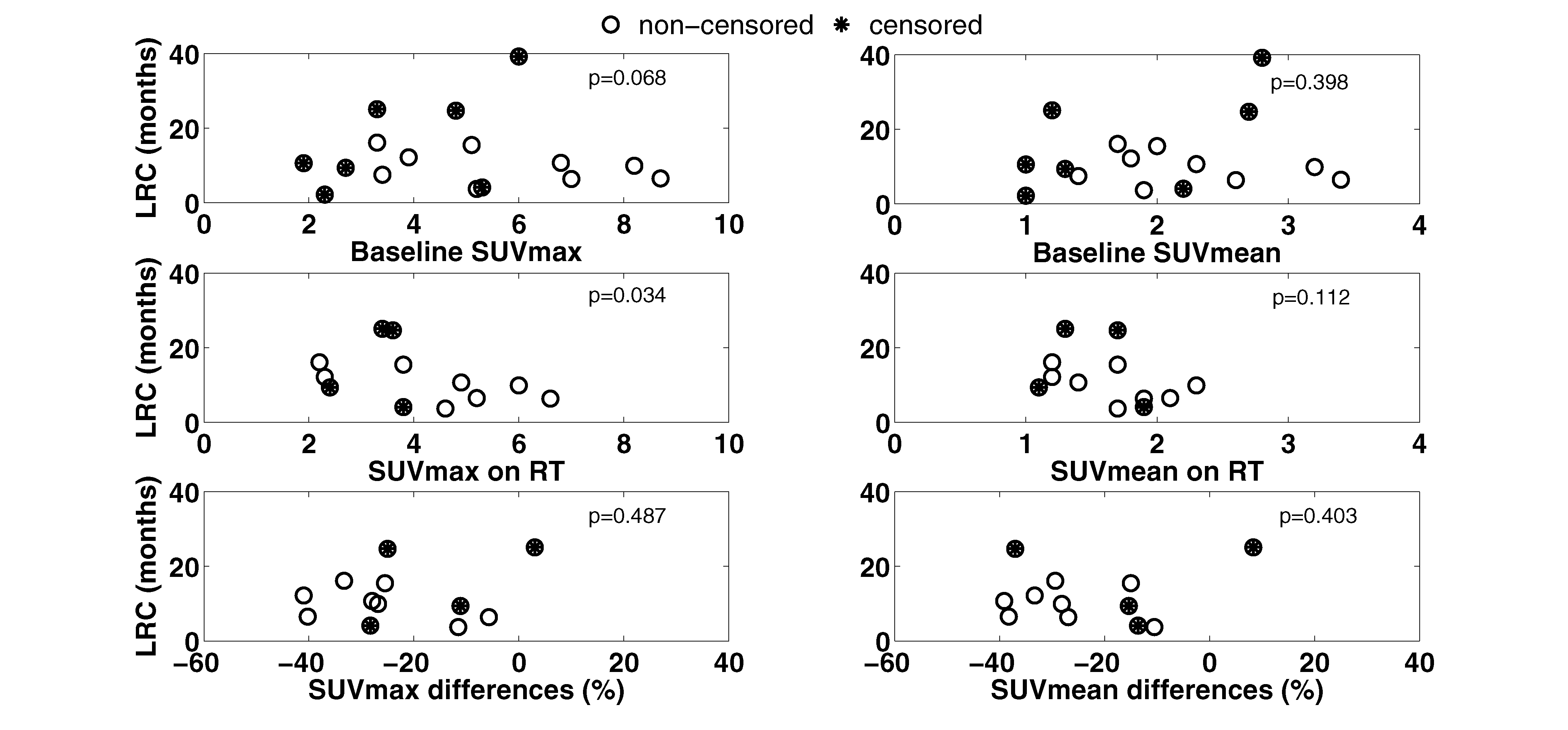

Supplement: Supplementary file 1 — Primary tumour locoregional control (LRC) plotted against primary tumour baseline SUVmax (left top), or SUVmean (right top), on-RT SUVmax (left middle), or SUVmean (right middle) and relative SUVmax (left bottom), or SUVmean response (right bottom). Univariate Cox regression model p values are attached to each subfigure. Empty and marked circles represent tumours which progressed and censored cases, respectively. (JPEG 699 kb) [file 259_2013_2632_Fig5_ESM.jpg]
